# Beyond Risk Factors: Lessons From an Elder Mistreatment Prevention Intervention

**DOI:** 10.1093/geroni/igaf122.1424

**Published:** 2025-12-31

**Authors:** Kelly Marnfeldt, Susanna Mage, Susan Enguidanos, Zachary Gassoumis, Kathleen Wilber

**Affiliations:** University of Southern California Leonard Davis School of Gerontology, Los Angeles, California, United States; University of Southern California Leonard Davis School of Gerontology, Los Angeles, California, United States; University of Southern California, Los Angeles, California, United States; UTHealth Houston, Houston, Texas, United States; University of Southern California, Los Angeles, California, United States

## Abstract

Elder mistreatment (EM) remains a critical yet under-addressed public health concern, particularly among people living with dementia (PLWD). Since caregiver stress is strongly linked to EM risk, community-based prevention efforts have primarily focused on reducing caregiver burden, depression, and anxiety. However, empirical findings suggest that the pathways leading to EM are more complex. The Comprehensive Older Adult and Caregiver Help (COACH) intervention is one of the few rigorously tested programs that challenge these assumptions. Despite successfully reducing EM, COACH did not significantly impact the hypothesized risk factors, raising questions about the mechanisms underlying its effectiveness. To explore this, we conducted a retrospective case study using the intervention itself as the unit of analysis. Findings suggest that COACH’s impact may have stemmed from its ability to enhance caregivers’ capacity to navigate challenges in real-time. Three key components were identified as potential contributors to its success: (1) tailored emotional and practical support that helped caregivers sustain their role; (2) care navigation and resource provision that facilitated access to essential services; and (3) caregiver education that provided problem-solving strategies for managing difficult situations. While COACH was effective, the lack of an explicit theoretical framework makes it difficult to pinpoint which elements drove its success. Strengthening future interventions with a theory-driven approach could improve their adaptability and predictability across diverse settings. These insights can help guide the development of caregiver support programs that mitigate EM risk while ensuring interventions remain effective and scalable.

